# High-grade biliary intraepithelial neoplasia localized in the distal bile duct diagnosed via peroral cholangioscopy: A rare case report

**DOI:** 10.1097/MD.0000000000040993

**Published:** 2024-12-20

**Authors:** Naohiro Kato, Atsushi Yamaguchi, Hiroki Kamada, Shigeaki Semba, Yuji Teraoka, Takeshi Mizumoto, Yuzuru Tamaru, Tsuyoshi Hatakeyama, Hirotaka Kouno, Yoshiyuki Shibata, Sho Tazuma, Takeshi Sudo, Kazuya Kuraoka, Shigeto Yoshida

**Affiliations:** a Department of Endoscopy, NHO Kure Medical Center and Chugoku Cancer Center, Hiroshima Prefecture, Japan; b Department of Gastroenterology, NHO Kure Medical Center and Chugoku Cancer Center, Hiroshima Prefecture, Japan; c Department of Surgery, NHO Kure Medical Center and Chugoku Cancer Center, Hiroshima Prefecture, Japan; d Department of Pathology, NHO Kure Medical Center and Chugoku Cancer Center, Hiroshima Prefecture, Japan.

**Keywords:** bile duct cancer, biliary intraepithelial neoplasia (BilIN), cholangiocarcinoma, high-grade BilIN

## Abstract

**Rationale:**

Biliary intraepithelial neoplasm (BilIN) is characterized by a microscopically identifiable preinvasive neoplasm of the biliary tract. BilIN is rarely diagnosed intentionally and is often detected incidentally in surgical specimens obtained via surgical resection for other types of cancers. Herein, we report a rare case of high-grade BilIN localized in the distal bile duct.

**Patient concerns:**

A 67-year-old female patient presented with epigastric discomfort approximately 1 month earlier. The patient underwent a blood test on the previous day at a local hospital. Results revealed abnormal liver function. Thus, the patient was referred to our hospital for further examination and treatment.

**Diagnoses:**

Blood tests showed elevated hepatic and biliary enzyme levels. However, jaundice was not noted, and the patient’s tumor marker levels were normal. Contrast-enhanced computed tomography scan revealed dilatation of the common bile duct and diffuse wall thickening of the bile duct and gallbladder walls. Tissue samples obtained via bile juice cytology showed a poorly differentiated adenocarcinoma. However, the bile duct walls were thick, and the tumor location was unclear. Hence, peroral cholangioscopy was performed. Results revealed an irregular mucosal surface and low papillary protuberances in the lower bile duct. Based on these findings, the patient was diagnosed with distal bile duct carcinoma.

**Interventions:**

She underwent surgical resection.

**Outcomes:**

Histological findings showed high-grade BilIN with scattered low-grade BilIN in the surrounding area.

**Lessons:**

We present a rare case of high-grade BilIN in the lower bile duct that was detected without other types of cancers and diagnosed via peroral cholangioscopy.

## 1. Introduction

Biliary intraepithelial neoplasm (BilIN) is characterized by a microscopically identifiable preinvasive neoplasm of the biliary tract,^[1]^ and it was first described in 2005.^[2]^ It comprises noninvasive, flat, or papillary lesions that are confined to the gallbladder lumen or bile ducts. BilIN represents the process of multistep cholangiocarcinogenesis, and it is the biliary counterpart of pancreatic intraepithelial neoplasia.^[3]^ BilIN was previously classified as BilIN-1, BilIN-2, or BilIN-3 based on atypia degree and epithelial polarization. Recently, the World Health Organization proposed that BilIN should be instead classified as low- or high-grade.^[4]^ Low-grade BilIN, corresponding to BilIN-1 and BilIN-2, is characterized by mild cytoarchitectural atypia, high N:C ratio, hyperchromasia, and prominent nucleoli.^[4]^ High-grade BilIN, corresponding to BilIN-3, is categorized as carcinoma in situ (Tis) and is characterized by complete loss of polarity, marked nuclear atypia, and frequent mitoses resembling micropapillae or tall papillae. BilIN is rarely diagnosed intentionally and generally found incidentally in surgical specimens obtained via surgical resection for other types of cancers.

## 2. Case presentation

**Figure 1.**
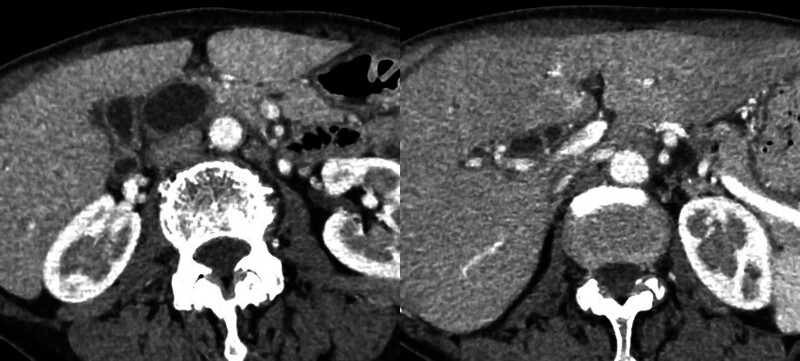
Contrast-enhanced computed tomography scan showing dilatation of the common bile duct without dilatation of the intrahepatic bile duct and diffused wall thickening of the bile duct and gallbladder walls.

**Figure 2.**
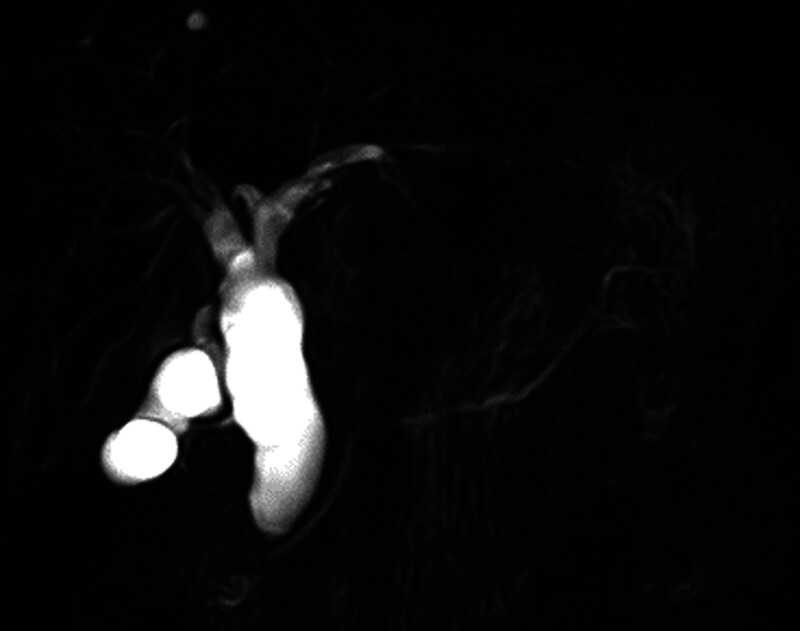
T2-weighted magnetic resonance cholangiopancreatography showing spindle-shaped dilatation of the common bile duct without lymph node swelling around the cystic duct.

**Figure 3.**
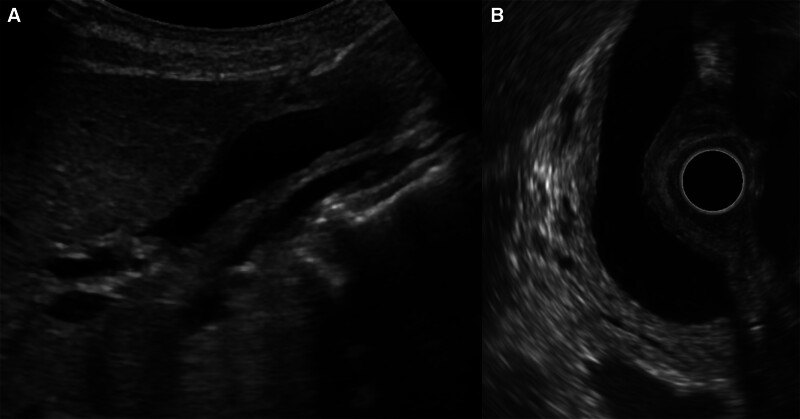
Abdominal ultrasonography revealing wall thickness of the right hepatic duct and upper bile duct (A). Endoscopic ultrasonography showing wall thickening up to the lower bile duct without evident tumor lesions (B).

**Figure 4.**
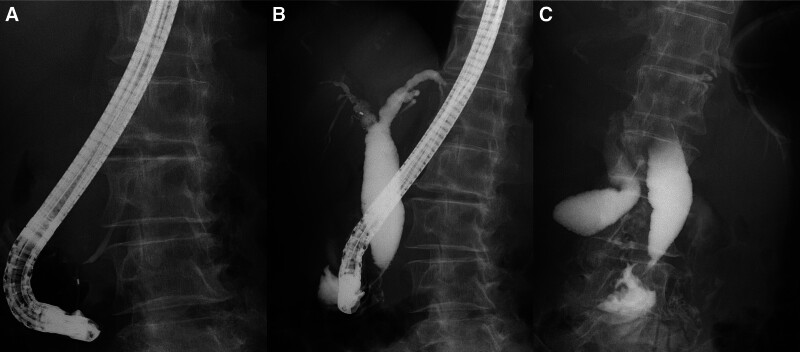
Endoscopic retrograde cholangiopancreatography showing a separate contrast imaging of the bile and pancreatic ducts with no indication of abnormal confluence.

**Figure 5.**
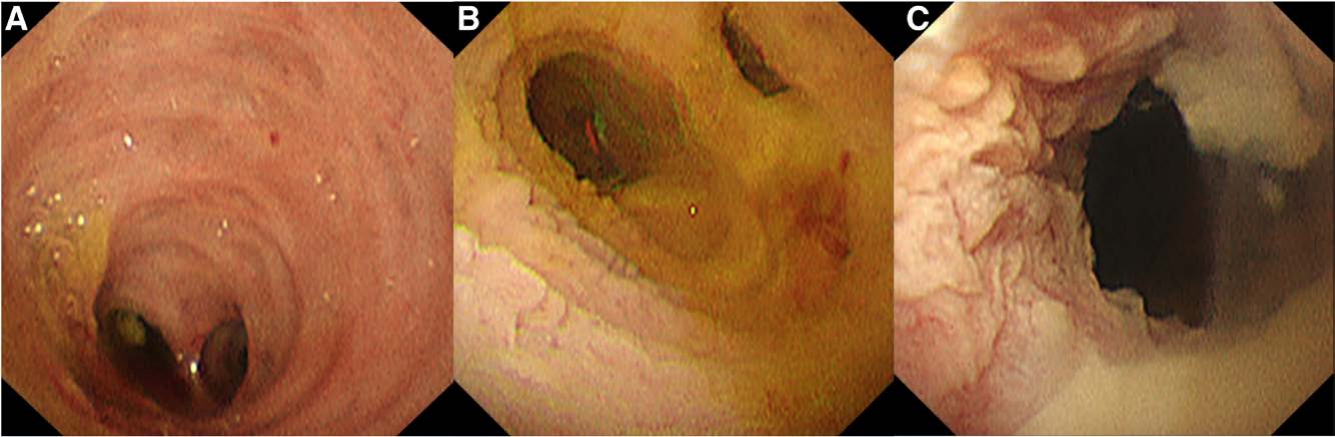
Peroral cholangioscopy revealing a whitish, rough mucosa in the upper to the middle bile duct (A) and (B), suspected as widespread fibrosis, and reddish irregular mucosa and low papillary protuberances in the lower bile duct (C).

**Figure 6.**
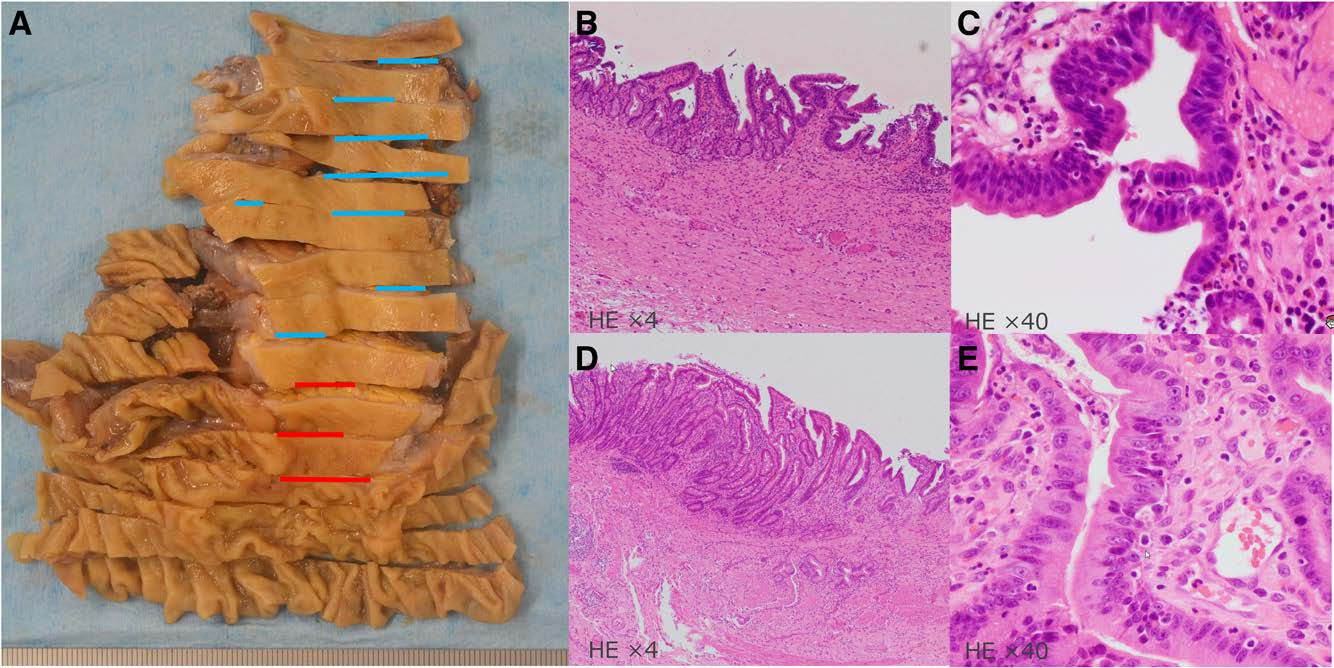
Macroscopic findings of the resected specimen revealing an irregular mucosa spreading 2 cm in the lower bile duct (A), and histological findings revealing atypical epithelium showing a partial loss of polarity, consistent with high-grade BilIN at the red line (D) and (E), with scattered low-grade BilIN in the surrounding area at the blue line (B) and (C).

A 67-year-old female patient presented with epigastric discomfort approximately 1 month earlier. The patient underwent blood test on the previous day at a local hospital. Results revealed abnormal liver function. Thus, the patient was referred to our hospital for further examination and treatment. Upon hospital visit, she did not have fever or other symptoms. Furthermore, she did not present with any significant occupational history (including work in the printing industry) or family history (including cancer). The patient’s medical history included hyperlipidemia. However, she was a nonsmoker, and drank little alcohol. Laboratory tests were performed using samples taken upon admission. Results revealed elevated hepatic and biliary enzyme levels without jaundice, and the levels of tumor markers, including carcinoembryonic antigen and carbohydrate antigen 19-9 were normal. Contrast-enhanced computed tomography (CT) scan showed dilatation of the common bile duct without dilatation of the intrahepatic bile duct. Moreover, diffuse wall thickening of the bile duct and gallbladder walls was observed (Fig. 1). However, no lymph node swelling was observed around the biliary tract. T2-weighted magnetic resonance cholangiopancreatography showed spindle-shaped dilatation of the common bile duct (Fig. 2). Abdominal ultrasonography revealed thickening of the right hepatic duct and upper bile duct wall (Fig. 3A). Endoscopic ultrasonography revealed wall thickening up to the middle and lower bile duct. Nevertheless, no evident tumor lesions were found (Fig. 3B). Endoscopic retrograde cholangiopancreatography showed a separate contrast image of the bile and pancreatic ducts, and there were no findings indicative of an abnormal confluence (Fig. 4A and B). In addition, the patient’s bile amylase concentration was normal (14 U/L). Cholangiography showed spindle-shaped dilatation of the common bile duct. However, the outflow of bile juice was adequate (Fig. 4C). Intraductal ultrasonography in the bile duct using the guidewire revealed thickening of the common bile duct and intrahepatic bile duct wall, with a well-maintained high-echoic outer layer. Fluoroscopy-guided biopsy of the upper, middle, and lower bile duct showed no malignant findings. Nevertheless, cytology specimens obtained from the common bile duct revealed a poorly differentiated adenocarcinoma. However, the bile duct walls were thick, and the tumor location was unclear. Hence, peroral cholangioscopy (POCS) was performed for further examination. POCS was conducted via cholangioscopy (CHF-B290 EVIS LUCERA ELITE; OLYMPUS) with sideview endoscopy. Results revealed a whitish and rough mucosa in the upper to the middle bile duct (Fig. 5A and B), suspected as widespread fibrosis, and a reddish irregular mucosa and low papillary protuberances in the lower bile duct (Fig. 5C). These findings led to the diagnosis of this causative lesion. Consequently, the patient was diagnosed with distal bile duct carcinoma, and she underwent pancreaticoduodenectomy. Thereafter, she was discharged and had an uneventful recovery. Macroscopic findings of the resected specimen revealed an irregular mucosa spreading 2 cm in the lower bile duct (Fig. 6A), and histological findings revealed an atypical epithelium showing partial loss of polarity, consistent with high-grade BilIN (Fig. 6D and E), with scattered low-grade BilIN in the surrounding area (Fig. 6B and C). Widespread chronic inflammation and fibrosis were observed in the background bile duct. No metastases were found in any of the resected lymph nodes (pTisN0M0, stage 0 in the Union for International Cancer Control TNM classification). The patient has been alive for 1.5 years after the surgery without recurrence.

## 3. Discussion

Herein, we present a patient with high-grade BilIN localized in the bile duct that was identified before surgery.

BilINs are commonly incidentally identified during examination or bile duct resection for other cancers or biliary tract cancers. This is because BilIN is flat or low papillary and difficult to identify on CT scan, ultrasonography, and even endoscopic ultrasonography. Thus, there are only a few case reports on independently operated BilIN.

We searched for case reports on high-grade BilIN from 1987 to 2023 in Igaku Chuo Zasshi and Pubmed using the following keywords: “high-grade BilIN” or “BilIN-3.” Results revealed high-grade BilIN cases,^[5–11]^ and 8 cases including our case were reviewed (Table 1). In these cases, 3 patients, including our patient, had only BilIN lesion, and 5 patients had other types of lesions (e.g., cholangiocarcinoma and gallbladder carcinoma). Five of the 8 patients showed no abnormality on images at the area of high-grade BilIN. However, 3 of the 8 patients exhibited bile duct wall thickening on CT scan, which is a vital indicator of high-grade BilIN and biliary malignancies.

**Table 1:** Case reports of high-grade biliary intraepithelial neoplasia.

Case	Ref.	Age/sex	Localization	CT/US feature	POCS feature	Accompanied lesion	Recurrence
1	5	62/M	Cystic duct	None	Not performed	Adenoma in bile duct	None
2	6	61/M	Distal bile duct	Wall thickness and stenosis of bile duct	Not performed	None	None
3	7	81/F	Distal bile duct	None	Not performed	IPMN with invasive carcinoma	Not available
4	8	69/M	Peri-hilar bile duct	None	Reddish granular mucosa	MANEC in bile duct	Recurrence of MANEC in 5 mo
5	9	84/M	Cystic duct	Wall thickness of bile duct	Reddish granular mucosa, dilated tortuous vessels on the mucosa	None	None
6	10	68/M	Distal bile duct	None	Not performed	Cystic duct carcinoma	None
7	11	70/F	Right intrahepatic bile duct	None	Not performed	Intrahepatic bile duct carcinoma	None
8	Our case	67/F	Distal bile duct	Wall thickness of bile duct	Reddish irregular mucosa, low papillary protuberance	None	None

Abbreviations: CT = computed tomography, F = female, IPMN = intraductal papillary mucinous neoplasm, M = male, MANEC = mixed adenoneuroendocrine carcinoma, POCS = peroral cholangiography, Ref = reference number, US = ultrasonography.

POCS was performed in 3 cases, and low papillary protuberances were also observed in these cases. Thus, the POCS findings may be useful for high-grade BilIN diagnosis. For the diagnosis of malignancy, the sensitivity and specificity in a pooled analysis with visual impression using a cholangioscopy were 84.5% and 82.6%, respectively. Additional cholangioscope-directed biopsies have a higher specificity.^[12]^ However, in our case, cholangioscope-directed biopsy could not be performed due to technical difficulties. In our case, the pathological result of the resected specimen was high-grade BilIN. However, preoperative bile duct brushing bile cytology was believed to be poorly differentiated adenocarcinoma. The reason why we considered that the lesion with nuclear atypia equivalent to high-grade BilIN was modified by crushing or other techniques during specimen collection.

Furthermore, we considered the background of carcinogenesis in this case. On histopathological examination, diffuse chronic inflammation was observed in the background of the resected bile duct, and low-grade BilIN was scattered around the high-grade BilIN and common bile duct, indicating long-term inflammation of the bile duct. Histologically, the high-grade BilIN lesion was not the cause of biliary obstruction, and there were no other abnormalities, such as bile sludge and stones, that could cause biliary obstruction. Therefore, in this patient, we considered congenital biliary dilatation (CBD) as the possible cause of carcinogenesis. The Todani classification is generally used for CBD. In most cases, CBD is associated with pancreaticobiliary malfunction (PBM), which is a risk factor of biliary cancer.^[13]^ In our case, the patient presented with CBD without PBM, which is classified as Todani type Ib. In fact, when we searched for cases of biliary cancer in CBD without PBM in Igaku Chuo Zasshi and Pubmed, there were 7 reported cases (Table 2).^[14–20]^ The mechanism of carcinogenesis in CBD is attributed to pancreatic juice reflux into the bile duct, damage to the bile duct wall associated with PBM, and the presence of genetic abnormalities.^[21,22]^ In the process of carcinogenesis in CBD associated with PBM, a histopathological hyperplasia–dysplasia–carcinoma sequence exists, and it is believed that carcinogenesis occurs due to repeated damage and regeneration of biliary mucosa.^[23,24]^ There is no pancreatic juice reflux into the bile duct and no evidence that the carcinogenic frequency is high in CBD without PBM. We suspected that continuous bile pooling after sludge formation and inflammation may lead to carcinogenesis. Further case studies are required to validate the mechanism of carcinogenesis in CBD without PBM. In the present case, managing the patient’s biliary carcinogenesis in the future is vital as her excided bile duct had several BilIN lesions, and the remnant bile duct must have several BilIN lesions.

**Table 2:** Case reports of cholangiocarcinoma of the common bile duct with congenital bile duct dilatation without pancreaticobiliary maljunction.

Case	Ref.	Age/sex	Todani type	History of surgery	Localization	Symptom	Tumor factor	Recurrence
1	14	39/F	Ib	Cystectomy and hepatocholangiojejunostomy (at 22 yrs old)	Remnant bile duct in pancreas	NA	NA	9 mo
2	15	53/F	N.A	None	Distal bile duct	Fatigue	T2	None
3	16	56/F	II	Choledochoduodenostomy (at 1 year old)	Lower bile duct	Back pain	T4	None
4	17	41/F	Ib	Cystectomy and hepatocholangiojejunostomy (at 28 yr old)	Remnant bile duct in pancreas	Upper abdominal pain	T4	None
5	18	53/F	II	None	Cyst in common bile duct	None	T4	None
6	19	37/F	N.A	None	Common hepatic duct	Upper abdominal pain	NA	None
7	20	54/F	V	None	Peri-hilar bile duct	Upper abdominal pain	T3	None
8	Our case	67/F	Ib	None	Lower bile duct	Epigastric pain	Tis	None

Abbreviations: F = female, NA = not available, Ref = reference number.

Taken together, the patient presented with high-grade BilIN, which is an extremely rare condition and difficult to diagnose before surgery. However, wall thickness of the bile duct may be an essential indicator, and POCS may aid in BilIN diagnosis.

## 4. Conclusion

Herein, we present a rare case of high-grade BilIN in the lower bile duct that was detected without other cancers and diagnosed via POCS.

## Author contributions

**Conceptualization:** Naohiro Kato.

**Data curation:** Naohiro Kato, Hiroki Kamada, Shigeaki Semba, Yuji Teraoka, Takeshi Mizumoto, Yoshiyuki Shibata, Sho Tazuma, Kazuya Kuraoka.

**Methodology:** Naohiro Kato, Atsushi Yamaguchi.

**Supervision:** Hirotaka Kouno, Shigeto Yoshida.

**Validation:** Yuzuru Tamaru, Tsuyoshi Hatakeyama, Kazuya Kuraoka.

**Writing – original draft:** Naohiro Kato, Atsushi Yamaguchi.

**Writing – review & editing:** Atsushi Yamaguchi, Takeshi Sudo, Shigeto Yoshida.
